# The Duogynon controversy and ignorance production in post-thalidomide West Germany

**DOI:** 10.1016/j.rbms.2021.09.003

**Published:** 2021-10-19

**Authors:** Birgit Nemec, Jesse Olszynko-Gryn

**Affiliations:** a Charité–Universitätsmedizin Berlin, Germany; b University of Strathclyde, Glasgow, UK

**Keywords:** Congenital malformations, Health activism, Ignorance production, Schering AG, Pregnancy testing, West Germany

## Abstract

This article examines the West German controversy over Duogynon, a ‘hormone pregnancy test’ and the drug at the centre of the first major, international debate over iatrogenic birth defects in the post-thalidomide era. It recovers an asymmetrical power struggle over the uneven distribution of biomedical knowledge and ignorance (about teratogenic risk) that pitted parent-activists, whistleblowers and investigative journalists against industrialists, scientific experts and government officials. It sheds new light on the nexus of reproduction, disability, epidemiology and health activism in West Germany. In addition, it begins to recover an internationally influential discourse that, in the post-thalidomide world, seems to have resuscitated antenatal drug use as safe until proven harmful.

## Between thalidomide and diethylstilbestrol

Between 1950 and the 1980s, millions of women worldwide found out whether or not they were pregnant by swallowing tablets or receiving injections. Duogynon, the first ‘hormone pregnancy test’ (HPT), was developed in the late 1940s by the West-Berlin-based pharmaceutical company Schering AG (acquired by Bayer in 2006). As a diagnostic drug, it ruled out gestation by inducing uterine bleeding (a ‘negative’ result); no bleeding confirmed pregnancy. When Duogynon debuted in the Federal Republic of Germany (FRG) in 1950, home pregnancy tests did not yet exist. Pregnancy diagnosis involved injecting laboratory animals – usually frogs – with a woman’s urine (Olszynko-Gryn, 2018b). At a time when laboratories were struggling to meet increasing demand (Olszynko-Gryn et al., 2018: 36), Schering marketed Duogynon as a cheaper and more convenient alternative to the expensive and cumbersome ‘frog test’ (Froschtest). Other companies followed suit, and a range of HPTs were marketed internationally (Olszynko-Gryn, 2014: 188–202), including through exclusive licencing agreements with Schering (Olszynko-Gryn, 2016).

Schering was one of a handful of large companies with in-house research laboratories that, in the interwar period, carved out a gynaecological market for industrial sex hormones (Gaudillière, 2018). Women’s bodies were increasingly ‘hormonalized’ as proprietary molecules proliferated in the medical management of menstruation, pregnancy and menopause (Malich, 2017, Oudshoorn, 1994). Duogynon contained norethisterone acetate, a synthetic progestogen used to prevent miscarriage, and ethinyl estradiol, a synthetic oestrogen used to treat symptoms of menopause. It was marketed simultaneously as a treatment for amenorrhoea (the absence of menstruation) and as a diagnostic test for early pregnancy. In 1961, Schering re-combined the same hormones in Anovlar, Europe’s first contraceptive pill.

The fact that Duogynon and Anovlar differed in dosage, regimen and indication but contained the same ingredients would link HPTs and oral contraception in the first major international debate over iatrogenic birth defects of the post-thalidomide era. The debate, which still resonates today, focused on the use and regulation of synthetic sex hormones in early pregnancy. It was sparked on 7 October 1967 by a brief report in the prestigious journal *Nature* (Gal et al., 1967). Lead author Isabel Gal, a paediatrician at Queen Mary’s Hospital for Children in Surrey, near London, UK, warned that Primodos – as Duogynon was called in Britain – might be causing spina bifida (a severe neural tube defect). She also implicated Amenorone Forte (Roussel Laboratories), the second most popular HPT in Britain, and Norlestrin (Parke-Davis), a contraceptive pill that, in the event of conception due to forgetfulness or some other reason, would likewise expose the fetus to norethisterone acetate and ethinyl estradiol (Marks, 2010: 77).

Like ‘the pill’ and other reproductive technologies, Duogynon was not one thing. Rather, the drug came in a variety of forms: ampoules (1950–1978); disposable syringes (1956–1980); and tablets (1958–1973). Tablets with a higher dose of norethisterone acetate became available in 1962. Sales figures indicate a turnover of 225,000 DM in 1952 (Schering, 1982). Twenty-five years later, Schering reportedly sold around 1.2 million units (ampoules and tablets) annually, valued at 3.3 million DM (Anon, 1977a, Anon, 1977c, Anon, 1978c). By then, cheap, reliable laboratory test kits as well as commercial home pregnancy tests had become available (Olszynko-Gryn, 2020), making it more difficult to justify the continued use of HPTs, and easier for Schering to remove Duogynon from the West German market in 1981. However, it did so only after sustained public pressure and legal action brought against the company by the young parents of ‘Duogynon-damaged children’.

HPTs faded from view in the 1980s, and there the story might have ended. Recently, however, a reinvigorated campaign – led by parents and their now-adult children in Britain and Germany – has once again drawn in journalists, scientists, politicians and lawyers (Brown et al., 2018, Cumberlege, 2020 Bundestag, 2021, Farrell and Lane, 2017, Farrell and Lane, 2020, Heneghan and Aronson, 2019, Heneghan and Aronson, 2021, Stücken, 2016, Henegahn et al., 2019). This time round, there is a huge amount of archival evidence to sift through, and historians have also become involved (Claes, 2020, Olszynko-Gryn et al., 2018, Weßel, 2018). So far, we know that some European countries – notably Norway, where a more precautionary attitude prevailed – took regulatory action earlier than others. We also know that in 1978, at a crucial juncture in the British debate, the UK Minister of Health used the negative results of a large West German study to block British calls for a public inquiry into the teratogenicity of HPTs (Olszynko-Gryn et al., 2018: 40). However, although HPTs originated in West Germany and remained on the market there for longer than anywhere else in Europe, we do not yet have a history of Duogynon in the FRG. Nor do we know much about the West German study that figured so decisively in the British debate.

This article examines the Duogynon controversy in post-thalidomide West Germany. As a contribution to the historical study of ignorance (Oreskes and Conway, 2010, Proctor and Schiebinger, 2008, Proctor, 2012, Tuana, 2006), it explores an asymmetrical power struggle over the uneven distribution of biomedical knowledge – and structured absences of knowledge – about iatrogenic birth defects. It also begins to connect the dots between thalidomide and diethylstilbestrol (DES), the second most infamous teratogenic drug after thalidomide. The first synthetic oestrogen, DES, gained notoriety in the 1970s as a transplacental carcinogen. It became a focal point of health activism and has attracted considerable historical attention, especially for the USA and France (Bell, 2009, Cody, 2008, Fillion and Torny, 2016, Fillion and Torny, 2021, Gaudillère, 2014, Langston, 2010). The Duogynon controversy, we argue, was pivotal in a larger and more international debate that centred first on thalidomide, then on synthetic sex hormones (including HPTs and DES), and later on a succession of other drugs including anticonvulsants and acne medications (Ferguson, 2021, Green, 1996, Heneghan and Aronson, 2019, Martin, 2017, Méréo, 2019, Sanders, 1998, Timmermans and Leiter, 2000). Reconstructing it, as we begin to do in this article, will enrich the historical understanding of (debates over) antenatal drug use and teratogenic risk in the post-thalidomide world.

## Teratology after thalidomide

The thalidomide disaster (1957–1961) structured all subsequent debates over iatrogenic birth defects, starting with the Duogynon controversy. Thalidomide was developed in the 1950s as a safer alternative to barbiturate sedatives by Chemie Grünenthal, a family-run company that made penicillin for the West German market. It debuted in West Germany in 1957 as Contergan, an over-the-counter treatment for morning sickness and all-round ‘wonder drug’. By the time it was discontinued, in 1961, it had damaged the limbs, face, eyes, ears, genitals and internal organs of some 5000 West German children, many of whom died young. The worldwide total of children born with malformations was at least 10,000, with many more deaths *in utero* (Johnson et al., 2018, Parle and Wimmelbücker, 2020, Vargesson, 2015).

The thalidomide disaster shocked a young republic emerging from an extended period of safety and prosperity. It was exacerbated in the FRG by high consumption of pharmaceuticals, lack of information about adverse effects, absence of effective drug laws or regulatory oversight, and poor communication between scientists and clinicians. West Germany was home to the world’s second largest pharmaceutical industry (after the USA), a booming domestic market for prescription drugs, and an industry-friendly government that held the medical profession and pharmaceutical industry in high regard (Daemmrich, 2002, Kessel, 2017, Kirk, 1999, Lenhard-Schramm, 2016, Lenhard-Schramm, 2017). From the late 1940s to the early 1970s, nation-building was synonymous with the West German ‘economic miracle’ (Uekötter, 2015: 81). The pharmaceutical industry, alongside the chemical and automotive industries, stood for national regeneration, wealth and trust. Thalidomide, however, precipitated a crisis of faith in medical authority and damaged patients’ faith in drug safety. In particular, those with access to international news sources came to suspect experts and regulators of routinely downplaying harm, such as the risk (to women, not fetuses) of breast cancer and fatal blood clots with Anovlar (Kessel, 2019).

Starting in the early 20th century, a number of environmental factors, including maternal infection, fetal anoxia, malnutrition, radiation, chemicals and pharmaceuticals, came under investigation as possibly teratogenic (Dron, 2016, Kalter, 2003). Suspicion fell on the synthetic sex hormones, with a consensus forming in the 1950s that progestogens such as norethisterone acetate could ‘masculinize’ the female fetus (Dubowitz, 1962, Schardein, 1980, Zander and Müller, 1953). Duogynon was first discussed as a possible teratogen by an expert commission of the Düsseldorf health administration following the withdrawal of thalidomide (T. Arndt quoted in Ulrich, 2016). However, despite the known risk of masculinization, sex hormones (including DES) were generally regarded as clinically safe (Langston, 2010: 95–96). As with other ‘undone sciences’ (Frickel et al., 2010), teratology, the science of birth defects, was allowed to languish despite (or because of) interest from social movements and civil society.

The already insecure status of teratology in the early FRG was further weakened by a post-war political agenda that favoured industrial research and development, including projects connected to nuclear power, and tended to side-line research into the environmental causes of congenital malformation (Kirk, 1999). Nor did the continuation of eugenic approaches to reproduction and more internalist or ‘hereditary’ explanations for disability after 1945 favour teratology as a science worthy of investment (Herzog, 2018, Rübsamen and Leder, 1955). Infectious disease and malnutrition, not drugs, dominated the post-war discourse in paediatrics, the clinical science most directly concerned with the immediate effects of congenital malformation. This changed only as a result of thalidomide, and the introduction of a more social approach to paediatrics from the mid 1960s (Spranger, 2016: 5).

## The German research foundation study

In the aftermath of thalidomide, individuals and families formed interest groups to challenge a *status quo* that perpetuated ignorance by keeping iatrogenic birth defects off biomedical research agendas (Nemec and Moser, 2017, Osten, 2011). Decision makers in politics, biomedicine and industry responded by agreeing to spend 21 million DM on a prospective clinical study on the ‘course of pregnancy and child development’ (DFG, 1963, DFG, 1977). Coordinated by the German Research Foundation (DFG), the multi-site, computerized study was exemplary of Cold War ‘big data’ projects (Aranova et al., 2017). It adapted the influential ‘risk factor’ model of epidemiology that had been pioneered in the USA in the 1950s (Patel, 2012, Timmerman, 2012). In addition, to generate practical knowledge about teratogenic risk factors, including drugs, it would monitor no fewer than 14,800 women and their children between 1964 and 1973.

DFG selected Siegfried Koller, formerly a leading Nazi-era biostatistician, to co-design the study and handle the patient data it would generate (Aly and Roth, 2004, Schappacher and Oehler-Klein, 2007). DFG also coordinated data collection at participating gynaecology and children’s clinics around West Germany, in cooperation with pharmaceutical companies and consulting institutions, including the US National Institutes of Health (NIH) and the World Health Organization. Gynaecologists collected information about maternal serum and hormone levels, general living and work conditions, diet and drinking habits, medication and vitamin consumption, household and garden chemicals, and other socio-economic and environmental factors. Paediatricians collected health data on newborns and toddlers (up to the end of the third year of life). Pathologists carried out cytogenetic examinations of stillborn embryos and placentas, as well as post-mortem autopsies of children who died before their second birthday. Koller used these datasets to investigate correlations between maternal conditions, on the one hand, and infant and child health, on the other.

The DFG study was modelled on the NIH-funded Collaborative Perinatal Project (1959–1965), which itself had looked to earlier British cohort studies (Hardy, 2003: 304). Part of a broader trend that promoted biostatistics centrally in US health research (Patel, 2013), the NIH project collected data on over 55,000 pregnant mothers and their children at 12 sites across the USA. It generated computer tapes equivalent to 6 million punched cards (Niswander and Gordon, 1972: 18), and hundreds of publications over a period of decades (Broman, 1984). It identified, amongst other findings, a link between maternal smoking and ‘cot death’ (sudden infant death syndrome) (Klebanoff, 2009). The DFG study similarly advised against the consumption of alcohol, cigarettes and (unspecified) medications in pregnancy, and recommended a general expansion of antenatal surveillance ‘from the beginning to the end of pregnancy’ (DFG, 1977, Oakley, 1984). It also produced a general list of ‘dangers to women who elude a sensible lifestyle’ (DFG, 1977: 7). Neither the NIH project nor the DFG study disclosed any new teratogenic drugs.

From 1964, however, the DFG study group had discussed the possible teratogenic risk of Duogynon in light of an observed correlation with birth defects (DFG, 1977: 29, 56). Individual researchers on the project warned against the antenatal use of sex hormones (Hartl, 1961, Döring, 1971). However, the concluding remarks of the report omitted these concerns (DFG, 1977: 7). Others, meanwhile, remarked on methodological issues that seemed to compromise the results of the study (DFG, 1977: 16–17).

For one thing, the analysis excluded women with ‘existing gynaecological illnesses’ (a rather broad category) and those who continued to take prescription medications in pregnancy. This accounted for 59% of pregnancies, of which a large number involved the use of an HPT (DFG, 1977: 7, 11, 16–17, 53, 56). For another, 30% of the pregnancies were monitored only after 12 weeks of gestation (DFG, 1977: 11). Many clinical reports ‘could not be accurately gathered’ (DFG, 1977: 17), so researchers instead relied on maternal memories (DFG, 1977: 53). Finally, Koller and the statisticians who processed the data explained several of the observed ‘associations’ not as causal relations but as ‘pseudo-correlations’ (DFG, 1977: 56). For example, pregnant women who took hormones or tranquilizers in early pregnancy showed a higher risk of early miscarriage. However, because the pharmaceuticals were administered to treat ‘another risk’ (‘uterine bleeding’ or ‘impending miscarriage’), the correlation was interpreted as a ‘logical association’ and the drugs were not implicated as a risk factor (DFG, 1977: 326).

Post-thalidomide cultures of antenatal drug use have not been examined historically for any nation. Despite the shock of thalidomide, antenatal prescribing and self-medicating practices seemed to persist more or less unchanged well into the 1970s (Wicklund, 1982). In Britain, for example, a landmark report on ‘injuries to unborn children’ in 1974 found that 82% of pregnant women consumed prescription drugs and 65% self-medicated (Law Commission, 1974: 7). The picture is similar in West Germany, where Koller reported that 75% of pregnant women surveyed for the DFG study had used medication in the first trimester alone, with ‘female sex hormone’ (without further specification) as the largest drug category (Koller, 1983: Table 3.5.3–5). As such, it is surprising that his final report devoted only six of 355 pages to ‘medication’ (Koller, 1983: 49–55). Also unclear is why the study failed to account for the ‘mode’, ‘dose’, ‘amount’ or ‘timing’ of the administration of medication (Koller, 1983: 50), despite a consensus that these were key factors in the teratogenicity of drugs (Grebe, 1955, Kalter, 2003, Rageth, 1959). For a project motivated by thalidomide, it was remarkably incurious about medicines.

## Blowing the whistle on Schering

Koller and his colleagues on the DFG study communicated its null results, without comment on conspicuous absences or methodological issues, well into the 1980s (Degenhardt, 1972, Koller, 1972, Koller, 1974, Koller, 1983, Michaelis et al., 1983). Others, however, took a more critical approach. Soon after the publication of Isabel Gal’s report in *Nature* in 1967, Ulrich Moebius, an employee of Schering Berlin, defected to become West Germany’s first pharma-critical activist. From 1963 to 1966, the trained medical doctor had recommended Duogynon to gynaecologists as a sales agent in Austria, Ireland and Switzerland. Then, in 1967, he learned about potential risks from the British debate and also saw that his colleagues ‘suspected the risks […] but denied in public that they did’ (quoted in Haarhoff, 2016). The Contergan trial (1968–1970), a highly mediatized criminal procedure against Grünenthal employees, kept the risk of iatrogenic birth defects in the public eye long after thalidomide had been discontinued (Bösl, 2014, Steinmetz, 2003, von Schwerin, 2009). Shortly after it ended, with a settlement and a verdict of not guilty, Moebius founded the surveillance initiative ‘Arzneimittelradar’ (‘Drug Radar’), and the journal *Arznei-Telegramm*.

Moebius worked to democratize biomedical knowledge that, as he saw it, paternalistic experts, corporations and regulators intentionally withheld from patients and consumers, to the detriment of public health. He published the first German warning against HPTs in *Arznei-Telegramm* in 1971 (Moebius, 1971: 38). However, his interventions remained largely unnoticed until 1977, when *Der Spiegel*, a leading weekly news magazine known for investigative journalism and breaking scandals, covered HPTs as part of a series of feature articles on the health risks associated with oral contraception (Anon, 1975, Anon, 1977b). Contributing to a public debate sparked by emerging feminist concerns with women’s health (Brot und Rosen, 1972), the magazine belatedly publicized the warnings of Gal and Moebius, and also alleged that the German whistleblower had been silenced for years (Anon., 1977a,c).

In contrast to the federal-government-centred approach favoured by the USA, the FRG vested pharma-regulatory authority in the medical profession and its self-governing associations (Daemmrich, 2002, Daemmrich, 2004). In the Contergan trial, the Federal Ministry of the Interior cooperated with medical experts to control the flow of information, excluding parents from the decision-making process and keeping them in the dark (Lenhard-Schramm, 2016). Experts also objected directly to participatory initiatives. The German Medical Association (BÄK) denigrated the patient-plaintiffs’ fight for resolution as the ‘total’ prosecution (H.H., 1970: 892); a direct comparison to Nazi Germany’s ‘total war’ effort after 1943. Schering’s legal department aimed to ‘trouble Dr. Moebius wherever it is possible’ (quoted in Haarhoff, 2016), and the Federal Association of the Pharmaceutical Industry (BPI) took legal action against him periodically. Ultimately, Moebius was forced to declare bankruptcy and sell his house in Holstein, but not before publishing the *Transparency List for German Pharmaceuticals*, a 176-page manual available for only 20 DM (Anon., 1977a).

The final ruling in the Contergan trial shifted the burden of proof from the patient-plaintiff to the corporate-defendant, and it established (at least in theory) a new epistemic and regulatory regime of postmarket surveillance and risk management: ‘In case of doubt over the teratogenicity of pharmaceuticals, they have to be taken from the market; a final proof of causality […] was not required.’ Legal and medical experts added the following clarification for clinicians: ‘pharmaceutical companies must take preventive measures before the risk of a medication expected to be harmful was scientifically proven’ (Hess, 1972; Landesgericht Aachen, 1970). However, BÄK clarified immediately that it would not be responsible for verifying any of the information given by pharmaceutical companies regarding drugs and their potential adverse effects (Bundesärztekammer, 1972: 1010). In practice, not much changed.

In 1973, Schering declared in its internal newsletter that it had ceased marketing Duogynon tablets for pregnancy testing, but without reference to possible risks (Anon., 1973: 2). Years later, some experts maintained that the change of indication had obviated the need for an official warning (Nocke, 1978). However, others argued that the new directions had never reached doctors, pharmacists or patients (Hammerstein, 1978: 1751). Journalists belatedly charged Schering with intentionally concealing information about Duogynon to shield Anovlar – widely used at that time by approximately 30% of women aged 15–44 (Sillies, 2010: 102–103) – from negative publicity (Anon, 1978b, Paul and Lempke, 1978a). Correspondence in Schering’s archives suggests a corporate strategy for managing uncertain knowledge and maintaining ignorance; namely, to ‘follow only the pressure of the health administrative bodies’ and to ignore ‘any scientific evidence’, even if the data suggested risks in connection to HPTs (quoted in Haarhoff, 2016). Regulators, however, seemed satisfied with Schering’s pro-forma information reports and did not apply much pressure.

## Regulating HPTs

Only in 1978, after *Der Spiegel* publicized Gal’s and Moebius’s warnings, did a group of German medical experts formally comment on Duogynon. After discussing the application of steroid hormones in early pregnancy at their annual meeting, the German Society for Endocrinology’s Permanent Commission for Steroid Toxicology (SKSDGE) gave an opinion: ‘potential teratogen effects’ of female sex hormones could not be proven, but ‘because of simple laboratory methods available for pregnancy diagnosis there was no need for the antenatal application of estrogens and progestogens for this purpose’ (SKSDGE, 1978). SKSDGE communicated the statement to all German gynaecologists and general practitioners (GPs) as ‘important information’ regarding Duogynon as well as Schering’s Gravibinon, Proluton and Proluton Depot, all used to prevent miscarriage.

Despite its timidity, the statement drew regulatory bodies into the debate. Several weeks later, the Medical Association’s Drug Commission (AkdÄ) released the first official warning against the prescription of hormones in pregnancy; namely, that ‘malformations after treatment of pregnant women with female sex hormones’ led to the clear guideline: ‘No pregnancy test with oral estrogen-progestogen combinations!’ (AkdÄ, , 1978, Hammerstein, 1978). By then, an international consensus had formed against the antenatal use of synthetic sex hormones (including DES), and several countries – Sweden (1972), Norway (1973), Australia, New Zealand, the USA (1975) and the Netherlands (1977) – had banned the use of HPTs. Belgium, Finland and Britain would follow suit in 1978 (Claes, 2020, Olszynko-Gryn et al., 2018, Weßel, 2018). However, in the FRG, powerful interests worked against the practical application of the new interdiction.

Retrospective epidemiological data was, and still is, viewed by many as weak compared with the gold standard of randomized clinical trials (Berlivet, 2005, Timmermans and Berg, 2003). Unconvinced that correlations implied causation, some clinical gynaecologists expressed concern that AkdÄ’s warning against HPTs would spread unwarranted ‘worry’ and ‘confusion’ (Nocke, 1978). Referring to the prospective DFG study and to a smaller Göttingen study, they recommended ‘caution’ but emphasized that a ‘teratogen effect […] was unproven on the basis of a careful analysis of the available data’ (DGGG, 1978, Knörr et al., 1975).

To distance itself from both its Nazi past and its socialist rival, the German Democratic Republic (GDR), the FRG minimized state involvement in health care and limited access to potentially sensitive statistical data, including data on disability (Lindner, 2010, Madarász-Lebenhagen, 2013). Many Western countries intensified the surveillance of birth defects after thalidomide (Al-Gailani, 2014, Fairchild et al., 2007, Lee, 2021). In contrast, the FRG, traumatized by Nazi ‘euthanasia’ and amidst a process of reappraisal, firmly rejected malformation registries; another form of ignorance production. Experts lamented that without a monitoring system, they would be unable to detect teratogenic effects, even if a drug increased the risk for congenital malformation by a factor of 500 (Anon, 1978b, Neubert, 1978). When the Health Ministry (BMJFG) requested more information about SKSDGE’s opinion, the Federal Public Health Service (BGA) stated – on the grounds that retrospective evidence was inadequate – that HPTs had ‘not been observed’ to cause birth defects in West Germany (BMJFG statement, 7.08.1978, quoted in Lenhard-Schramm, 2018: n6). BMJFG took no further steps (Lenhard-Schramm, 2018).

New legislation, the Drug Law of 24 August 1976, came into effect in 1978, and created BGA’s Institute for Drugs to oversee drug testing as part of revised licensing procedures. In a climate of intensifying activism around consumer rights, environmental pollution and occupational health (Westermann, 2013), the Consumer Associations’ Committee (AGVV), a national umbrella organization to support consumer-citizenship rights, requested a total ban on HPTs (Anon., 1978c). However, after an expert roundtable and discussions in parliament (Deutscher Bundestag, 1978), (Deutscher Bundestag 1978) BGA only ‘discouraged’ the use of norethisterone acetate and ethinyl estradiol in pregnancy diagnosis (Bundesgesundheitsamt, 1978).

Schering was obliged to communicate the new guidance, but, as BGA’s successor, BfArM (Federal Institute for Drugs and Medical Devices), would later admit, there was ‘no evidence [in BGA’s archives] to say whether or not this happened’ (Haarhoff, 2010). Although BGA does not seem to have actively enforced its guidance, Schering’s records show that information was included in newsletters and with patient information leaflets. The company also removed the diagnostic indication for ampoules and changed the name of the tablets from Duogynon to Cumorit ‘to raise awareness for the new use’ (exclusively as a treatment for amenorrhoea) (Schering, 1978). Some journalists, however, viewed the rebranding as a cynical manoeuvre to protect a ‘contested drug’ from ‘slander’, and launched further investigations (Paul and Lempke, 1978a). Despite increasing pressure, Schering’s head of clinical research held fast: HPTs would remain on the West German market to protect the reputation of compositionally similar products, including Anovlar (Lachnit-Fixon, 1978).

## Off label

Duogynon/Cumorit, as we have seen, was not one thing. It contained multiple meanings and uses that flexibly accommodated changing regulatory regimes and legislation. In this sense, it was no different from other drugs over which physicians have historically exercised significant authority independent of regulatory oversight (Greene and Watkins, 2012, Marks, 2014). The US Food and Drug Administration (FDA), for example, regulated the pharmaceutical industry, but tended to leave physicians alone in deference to their professional autonomy (Carpenter, 2010, 617–621). Similarly, physicians in the FRG enjoyed considerable ‘therapeutic freedom’ (to prescribe medicines as they saw fit) and were not closely monitored by the state (Kessel, 2009).

As a result, many drugs have led ‘double lives’ (Anon., 1967); approved for one thing, but prescribed for something else entirely. Companies are sometimes accused of unethical marketing, as in the Mediator (benfluorex) scandal that rocked France in 2010 (Lellinger, 2014, Lellinger, 2018). However, in certain areas of medicine, including paediatrics, psychiatry, oncology and reproduction, ‘off-label’ prescribing is par for the course (Balan et al., 2018, Becker and Wilman, 2012, Bell and Richards, 2021, Gershon and Shorter, 2019, Saiyed et al., 2017). Particular classes of drugs, including hormones, have found large markets for unapproved indications (Hoberman, 2005, Sanabria, 2016). As children and women of childbearing age are excluded from clinical trials, they tend to be more exposed to untested treatments (Epstein, 2007). The situation is further complicated in reproductive medicine by legal grey areas or proscriptions regarding contraception and abortion that discourage manufacturers from applying for licences (Weeks et al., 2005).

Misoprostol, for instance, is indicated for ulcers, but is widely used informally both as an abortifacient (Löwy et al., 2020, MacDonald, 2020) and to induce labour (Towghi, 2014; Voigt et al., 2015). Soon after the FDA approved DES as a menopause drug, in 1941, physicians started prescribing it, off label, as an informal treatment for miscarriage (Bell, 2009: 17). Later, in the 1960s, it was further repurposed as an (unofficial but not illegal) postcoital contraceptive or ‘morning after pill’ (Foster and Wynn, 2012, Prescott, 2011). When the FDA approved Enovid as a menstrual regulator in 1957, it did so with the understanding that physicians would be prescribing it for contraception (Junod and Marks, 2002, Tone, 2002). We are still researching the marketing and prescribing practices around Duogynon, and a complex picture is beginning to emerge.

Historical evidence is scarce. However, the contraceptive or abortive use of HPTs has been reported anecdotally for the USA, Britain, Israel, Nigeria, Peru and South Korea (Anon, 1967, Bonnema and Dalebout, 1992, Cumberlege, 2020, Harlap et al., 1975, Ujah, 1991). In Britain, some experts privately suspected women of intentionally misleading their GPs (about the timing of their last period) to gain access to HPTs for the purposes of inducing miscarriage (Smithells, 1967). Isabel Gal herself speculated that HPTs were ‘frequently used to induce abortion’ (Gal, 1972: 167). This parallel conversation about HPTs tended to happen in private or between experts. In West Germany, however, it was broached publically in *Stern*, a glossy weekly entertainment and political magazine for young adults that had lobbied for abortion law reform in the early 1970s (Anon., 1971).

Postcoital contraception was not approved in the FRG until the early 1980s (Olszynko-Gryn, 2018a: 549). Although under development in Sweden from the 1960s (Ramsey, 2021), abortion pills would not be marketed in any country until 1988, and not in Germany until 1999, well after reunification. As such, in the late 1970s – after abortion law reform but in the absence of officially sanctioned options for postcoital contraception or pharmaceutical abortion – women and their doctors improvised what a pair of journalists writing in *Stern* magazine evocatively called ‘the Duogynon ritual’.

In 1978, shortly after Schering had removed the diagnostic indication for Duogynon, they reported that West German doctors had prescribed no fewer than 550,000 doses of the drug in a single year; a conspicuously large number next to the estimated 125,000 women suffering from secondary amenorrhoea each year. On the basis of available sources, it is difficult to confirm the discrepancy, or whether Schering discouraged, ignored or actively supported unapproved uses of its product to maintain sales. What matters here is that the claim was publicly debated and prompted critics to question the reproductive politics of Duogynon and the meaning of its popularity.

Many GPs, it seems, remained ignorant of the changed indication and potential risk, and so continued to prescribe Duogynon, in good faith, as a pregnancy test. Others reportedly prescribed Duogynon to ‘stall’ patients seeking an abortion; if the drug did not induce miscarriage, then the operation would be delayed, at greater risk to the patient (Wolff, 1978). Still others, *Stern* alleged, conspired with their patients to use Duogynon as a postcoital contraceptive or abortifacient (Paul and Lempke, 1978a). As with many other once-popular practices of birth control (Fisher, 2006, Jones, 2020), we may never know how effective Duogynon was at achieving the desired result. In Britain, however, experts privately agreed that HPTs ‘undoubtedly’ induced miscarriage (Hobson, 1966), especially in ‘not well established’ pregnancies (Warrack, 1966); they estimated that approximately 10% of ‘negative’ results were really abortions (Dean, 1968).

West German women who knowingly used Duogynon as a morning after pill or abortifacient tended to be well educated and middle class. For them, tactical ignorance about whether or not conception had occurred functioned as a resource. In this sense, the Duogynon ritual resembled the venerable practice of taking ‘female pills’ to induce ambiguous uterine bleeding and other practices of menstrual regulation (Murphy, 2012, Olszynko-Gryn, 2018a, Pavard, 2019, van de Walle and Renne, 2001). Many other women, however, had taken Duogynon at face value as a pregnancy test or treatment for amenorrhoea. A subset of these women had been pregnant at the time and, following the birth of a child with a heart defect or shortened limb, subsequently learned about the possible teratogenic risk in a newspaper or magazine. It was these mothers who publicly accused Schering, ‘irresponsibly and knowing doctors’ ignorance and prescribing habits, of contributing daily to the use of women as test objects and the burdening of their pregnancies with unimaginable fears’ (IGDGK, 1979).

## The challenge from parent-activists

As with their counterparts in other countries (Kline, 2010, Murphy, 2012, Olszynko-Gryn, 2019, Pavard, 2019), women’s health campaigners in West Germany challenged medical paternalism and promoted an alternative epistemology that stemmed from embodied knowledge (Tuana, 2006). Following the example of the Boston Women’s Health Book Collective (Davis, 2007), a group of women who had met in 1971 at a landmark abortion law reform demonstration in Berlin self-published *Frauenhandbuch Nr. 1*. The chapter on the pharmaceutical industry critiqued Schering’s monopoly on ‘ovulation inhibitors’, and indicted Duogynon not for causing birth defects, but for generating unconscionably high-profit margins due to state ignorance for regulation and safety: ‘Our abdomen: their best business’ (Brot und Rosen, 1972: 110). Feminist activism helped to bring about abortion law reform in 1976 (Ferree, 2018, Herzog, 2014). From the mid 1970s, it sustained a larger public debate on women’s reproductive health (Kuhlmann and Kolip, 2005, Nemec, 2020, Schulz, 2002, Silies, 2010). However, direct resistance to Duogynon as a possible teratogen came from elsewhere.

Around 200 parents formed the Interest Group for Duogynon-Damaged Children (IGDGK) in 1978. The group was initiated by Edeltraud Müller from Offenbach near Frankfurt, whose daughter Ursula was born with spina bifida; unlike in her previous pregnancies, the mother of seven had been prescribed Duogynon to ‘reactivate menstruation’ (Paul and Lempke, 1978b). The mothers and fathers who joined forces with Müller were not seasoned activists; they did not come together at a demonstration in Berlin, but rather found one another via newspaper appeals. In this sense, they resembled other interest groups that promoted awareness about specific conditions (Epstein, 2008, Lindner, 2004, Söderfeldt, 2020).

IGDGK demanded a ban on the ‘second Contergan’ and prepared to take legal action against Schering (IGDGK, 1979). Crucially, *Stern* supported the campaign with a series of in-depth reports that pictured the children – some with limb defects reminiscent of thalidomide, others with life-shortening organ damage – in their homes in West Germany (Neuwied, Pfronten, Waldrach, Wanne-Eickel) and Britain (Liverpool, Manchester), where a similar campaign was underway. Two investigative journalists specialized in sexual and reproductive health, Klaus Lempke and Rainer Paul, gave a voice to mothers who had taken HPTs while pregnant, and to the parents of children who had died young.

With dramatic headlines (‘A Thousand Children Are Impeaching’; ‘Who Will Help Us?’; ‘Suspicion Hardened’), *Stern* amplified the charges that campaigners brought against Schering for intentionally withholding information. It pressed the case for regulatory failure and the uneven distribution of public knowledge about the possible risks of HPTs in different parts of the world (Anon, 1978d, Paul and Lempke, 1978a, Red., , 1979). In addition, it reported on a leaked confidential correspondence between Schering’s West German headquarters and its British subsidiary, Pharmethicals (Olszynko-Gryn, 2017). London medical directors had pleaded that it was Schering’s ‘moral duty’ to take Primodos off the market until the drug was proved ‘safe’. However, management in West Berlin maintained that the results of Schering’s in-house clinical research were “anything but alarming and we don’t see a reason to withdraw ‘Primodos’” (quoted in Paul and Lempke, 1978a). Concerned with the report, Schering hired Cologne attorneys to prohibit sales of the offending issue of *Stern* in Britain (Haarhoff, 2016). However, the warnings had already reached a large, international readership (Anon, 1978a, Kamke, 1978a, Kamke, 1978b). In the following months, the charges shifted to court.

Armed with animal evidence of teratogenicity dating back to 1969 (Setsevits, 1981), IGDGK initiated legal action against Schering in August 1978 (Dudda, 1982). The defence counsel responded by enlisting scientists, including Koller, as expert witnesses. From a statement by the team, we know that, in a preparatory meeting in October 1978, Koller proposed dismissing patient testimonials regarding adverse effects (Setsevits, 1981: 24–26). This is consistent with his previous support for discriminatory practices against vulnerable groups as the leading biostatistician under National Socialism (Schappacher and Oehler-Klein, 2007). Koller was generally hostile towards parent-campaigners and enjoyed ‘amicable relations’ with Schering and the BGA (Setsevits, 1981: 24). As an influential, well-networked expert, he was able to exploit established hierarchies to perpetuate the uneven distribution of biomedical knowledge and ignorance about iatrogenic birth defects in the FRG; a young nation where children in general, and people with disabilities (including children), were still only weakly enfranchised (Rudloff, 2002: 402–409).

The public prosecutor’s preliminary investigation ended after 2 years, on 19 December 1980, with a pre-trial decision that no negligent physical injury or offence against the drug law could have occurred because the unborn were not protected by the constitution of the FRG (Netzwerk Duogynon, 2019). Significantly, the decision came at a time of renewed antiabortion and disability rights activism (Herzog, 2018); we are still in the process of exploring the connections, but it may have come as a relief to defenders of the 1976 abortion law. Schering, meanwhile, removed Duogynon from the West German market in February 1981, later than in other Western countries. Rebranded as Cumorit, it remained available in India and other ‘developing’ nations well into the 1980s (Marcelis and Shiva, 1986, Silverman et al., 1992). Today, Congolese women still use unauthorized pills labelled ‘Duogynon’ for postcoital contraception (Hernandez et al., 2020); a telling hangover from the past. Norethisterone acetate and ethinyl estradiol continue to be found in contraceptives, treatments for endometriosis, and period delayers. However, the diagnostic use of sex hormones in early pregnancy is not coming back.

## Producing ignorance (about iatrogenic birth defects)

This article has examined the West German controversy over Duogynon, the original HPT and drug at the centre of the first major, international debate over iatrogenic birth defects of the post-thalidomide era. Sparked by Isabel Gal in 1967, the debate came to focus on the synthetic sex hormones, including DES. An international consensus formed in the 1970s that HPTs were unnecessary, correlated with birth defects, and should not be used in pregnancy. Crucially and in contrast to thalidomide and DES, however, the question of causation remained (or was actively kept) open in the case of Duogynon. The history of HPTs has much to teach about how teratology after thalidomide produced not only new knowledge, but also doubt, uncertainty and structured forms of ignorance (about iatrogenic birth defects). It also provides grounds for revisiting and reflecting on historical relationships between reproductive politics, civil society and disability in post-thalidomide West Germany.

In West Germany, the global epicentre of the thalidomide disaster, a powerful coalition of pharmaceutical industrialists, politicians and biomedical researchers exerted control over the production, circulation and reception of biomedical knowledge about birth defects. The perpetuation of ignorance about a possibly causal association between Duogynon and birth defects also benefited from nationally specific historical continuities regarding: established pathways in the organization of biomedical research; the high standing and political support enjoyed by the pharmaceutical industry as emblematic of national regeneration and economic growth; and National Socialism’s legacy of highly authoritarian power structures. An aversion to collectivist approaches in public health care (associated with the Third Reich and the GDR) further militated against the surveillance of birth defects in the FRG.

The DFG study, directed by Koller, was lauded in Britain as the ‘most comprehensive investigation ever conducted’ into iatrogenic birth defects (Olszynko-Gryn et al., 2018: 40). However, on closer inspection, surprising omissions come into view. As we have shown, the study was an exercise not only in the production of knowledge, but also in the perpetuation of ignorance. As if designed to generate a null result, it failed to collect crucial information, excluded the majority of patients from analysis, discarded data, and systematically interpreted significant correlations as non-causal – methodological issues that became apparent not only in retrospect, but were remarked on at the time.

It fell to parent-activists to challenge the negative findings as well as the *status quo* they upheld. Less powerful actors – the mothers and fathers of ‘Duogynon-damaged children’ – proved sufficiently resourceful to mobilize allies. Aided by whistleblowers, journalists and lawyers, they succeeded in shifting the arena of debate to the public sphere. Having forged new alliances, parent-activists redrew the borders of knowledge and ignorance (about iatrogenic birth defects). By bringing to light apparently supressed information, they seemed to expose something resembling a cover up (Stücken, 2016). Their legal action failed, but it generated a massive archive that may yet prove consequential. As an example of the active production of ignorance, the Duogynon controversy is typical of asymmetrical power struggles over the uneven distribution of biomedical knowledge – and structured absences of knowledge – that continue to this day. Not just another drug scandal, it was pivotal in a larger and more international debate that centred first on thalidomide, then on synthetic sex hormones (including HPTs and DES), and subsequently on a succession of other drugs.

## Declaration

The author reports no financial or commercial conflicts of interest.
